# Case report: A rare case of dual primary synchronous malignancies of the breast and kidney in a 70 year female

**DOI:** 10.1016/j.ijscr.2024.109400

**Published:** 2024-02-15

**Authors:** Tarbia Hamid, Fatima Khan, Nuzhat Sultana Khattak, Mian Naushad Ali Kakakhel, Ramsha Hamid

**Affiliations:** a Northwest General Hospital and Research Center, Passport Office Rd, Phase 5 Hayatabad, Peshawar, Khyber Pakhtunkhwa 25100, Pakistan; b Northwest School of Medicine, Plot #8, Sector A-2 Phase 5 Hayatabad, Peshawar, Khyber Pakhtunkhwa,25000, Pakistan; c Department of Community Medicine, Khyber Medical College, University of Peshawar, Rd No. 2, Rahat Abad, Peshawar, Khyber Pakhtunkhwa 25120, Pakistan

**Keywords:** Breast cancer, Renal cell cancer, Breast surgery, Synchronous primary cancers, Case report, Multidisciplinary team

## Abstract

**Introduction:**

Multiple primary cancers (MPCs) have attracted attention in medical research due to their increasing incidence. The coexistence of invasive breast carcinoma and clear cell carcinoma of the kidney, alongside a family history of cancer, highlights the multifactorial origins of MPCs, particularly their potential association with genetic factors.

**Case presentation:**

A 70-year-old female initially sought medical attention for a two-year history of a right breast lump as her primary concerns centered on the long-standing lump. Clinical evaluations and imaging studies revealed an invasive breast carcinoma diagnosis, and simultaneously, an incidental mass in her left kidney was identified as clear cell carcinoma.

**Discussion:**

Emphasis and further researh should be on the potential role of genetic factors in MPC development, necessitating comprehensive genetic evaluations.

**Conclusion:**

This study highlights the significance of customized treatment approaches for each malignancy, facilitating early detection, improved patient outcomes, and an enhanced understanding of MPCs.

## Highlights

• There is an increasing incidence in the rate of multiple primary synchronous tumors.
• Genetic and environmental factors contributing to development of multiple primary cancers need to be evaluated.
• Clinical presentation of primary breast cancer and primary renal cell carcinoma is very rare.
• Multidisciplinary treatment plan for synchronous tumors needs to be formulated and tailored to every patient.

## 1 Introduction

The phenomenon of multiple primary cancers (MPC) has intrigued the medical community for well over a century, dating back to Bill Roth's initial documentation in 1889.The widely accepted definition of MPC, as introduced by Warren and Gates, emphasizes that each neoplasm must represent a distinct malignancy, with a strict exclusion of a metastatic origin; as a. Histological confirmation of malignancy in both the index and secondary tumors. b. There should be at least 2 cm of normal mucosa between the tumors. If the tumors are in the same location, then they should be separated in time by at least five years. c. Metastatic probability of each other must be excluded [1]. There has been ongoing variation in the terminology used to describe MPCs, including terms like synchronous, simultaneous, metachronous, or successive neoplasms. These terms are centered on when neoplasms are discovered, rather than when the disease initially manifests. Synchronous malignancies, characterized by the concurrent emergence of one or more tumors either occurring simultaneously or manifesting within a period of six months, while ‘metachronous’ denotes the identification of a distinct neoplasm in a patient who already has a neoplasm (a successive neoplasm) [2,3]. While breast and renal carcinomas are well-documented entities individually, their concurrent presentation in a single patient is exceptionally rare, highlighting the uniqueness of this case. To our knowledge only 11 cases have been reported prior to this [4,5]. This rarity emphasizes the need for a meticulous approach to diagnosis, intervention, and ongoing care. We present this case which serves as a poignant reminder of the need for continued research in the field of oncology to address rare and complex presentations, such as the one presented here. This case report has been reported in accordance with SCARE guidelines [6].

## 2 Presentation of case

**Fig. 1:**
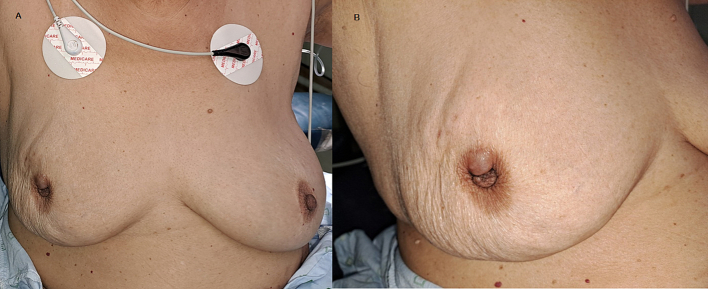
Preoperative breast images. A- Bilateral breasts. B - Right breast.

A 70 years old female, mother of seven children, presented to the OPD with the chief complaint of a right breast lump since the last two years. Her medical history included hypertension, diabetes mellitus, and a family history of cancer (mother had lung cancer). She had breastfed all her children, and was a non-user of oral contraceptives (OCPs). Upon general examination, she was very frail and had a BMI of 19. On inspection an asymmetry of the right breast was evident; there was a swelling in the upper outer quadrant with an overlying bulla. The Nipple was retracted, but had no visible discharge (Fig. 1). On palpation, there was a lump at 8'oclock to 9 o'clock, roughly measuring 4x3cm just above the nipple. It was hard, mobile over underlying muscles but adherent to the overlying skin. This was accompanied by palpable ipsilateral axillary lymph nodes. The examination of the left breast and axilla was unremarkable.

**Fig. 2:**
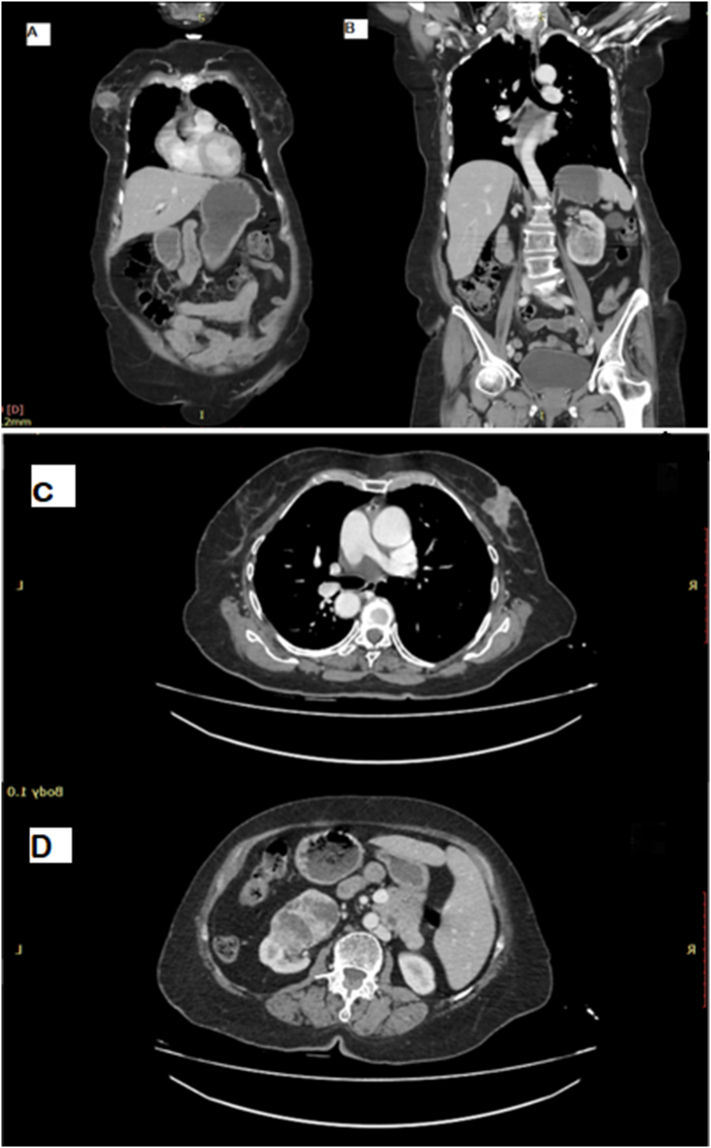
CT Chest abdomen and pelvis (axial views) showing right breast Ca (A) and left renal Ca (B) coronal views showing right breast Ca (C) and left renal Ca (D).

A diagnostic breast ultrasound (US) revealed a hypoechoic mass within the right breast in the retro-areolar region with posterior shadowing. The mass measured 3.8 cm × 2.5 cm in size. Architectural distortion in areas adjacent to the mass with soft tissue edema was seen. Ultrasound (US) of both mammary glands was suggestive of malignant looking mass in right mammary gland and a normal parenchymal echo pattern of the left breast. Staging Chest Abdomen and Pelvis CT scan showed a right breast mass with right axillary lymphadenopathy. Core biopsy of the main lesion and fine needle aspiration cytology (FNAC) of the axillary mass were done under image guidance by the Interventional radiologist on board. Multiple enlarged lymph nodes measuring up-to 1.0 × 0.8 cm were seen in the right axilla with loss of central hilum and increased cortical thickness. US guided FNAC of axilla showed atypical cells. Histopathology confirmed invasive ductal carcinoma grade two (!DC-2) of the right breast. Immunohistochemical stains revealed ER + ve/PR + ve and Her 2 neu Negative receptor studies. CT scan also revealed a large necrotic lesion/mass seen on left kidney (Fig. 2). Biopsy of the renal mass was also done under image guidance and histopathology showed clear type renal cell carcinoma, grade 2.

**Fig. 3:**
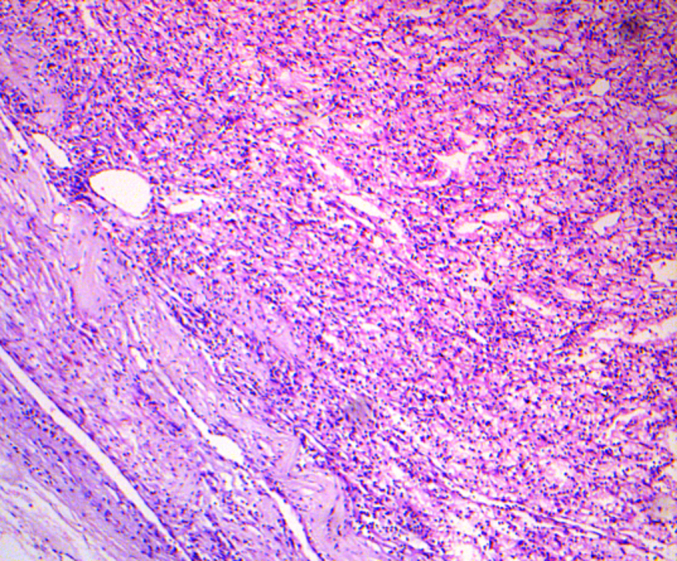
Histopathology sections from the renal mass of patient showing clear cell renal carcinoma.

**Fig. 4:**
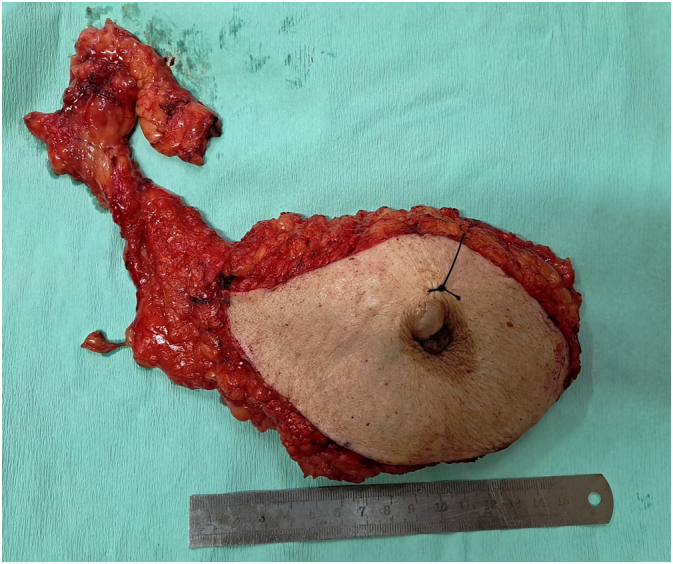
Resected modified radical mastectomy specimen of the Right breast with axillary dissection.

**Fig. 5:**
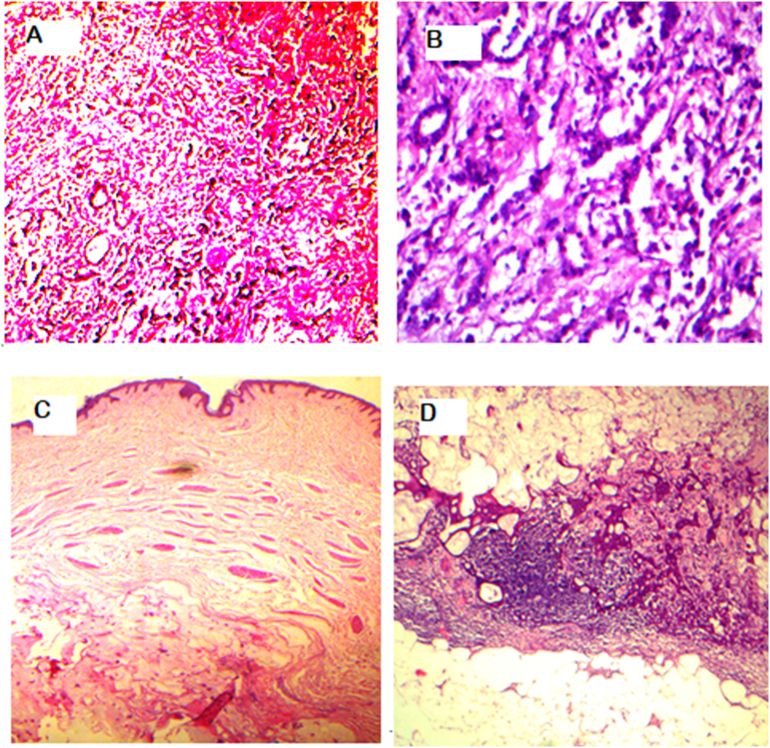
Invasive ductal carcinoma magnification 10 x (A) Gland formation at 40 x magnification in grade II invasive breast carcinoma (B) Dermis in skin of nipple and areola and lymph nodes are involved by invasive breast carcinoma with mucinous features at 10 x magnification (C, D).

While awaiting the multidisciplinary team meeting decision regarding, neaoadjuvant chemo and whether the patient would be fit enough to endure it, it was decided that the nephrectomy be done as soon as possible. The urologist on board was involved and a radical nephrectomy was done. Histopathology revealed a tumor size of 6 × 4 × 3.7 cm, in the lower pole of left kidney, and extent remained limited to the left kidney (Fig. 3). Pathological staging was pT1b, pNx, pMx. A staging bone scan showed no definite evidence of skeletal metastases. Surgical interventions were tailored to address each malignancy and following left nephrectomy in May 2023 a modified radical mastectomy was performed in August 2023 as she was deemed unfit by the multidisciplinary team for chemotherapy. Mastectomy specimen showed a main lesion of 3 × 2.8 × 2.5 cm, IDC-2 with mucinous features, all margins being tumor free (Fig. 4). 9/20 lymph nodes removed were positive for metastasis with extra-nodal extension. Pathological staging was pT2, N3a, pM0 (Fig. 5). Post-operatively, she received 15 cycles of radiotherapy for loco-regional control and was put on aromatase inhibitors. She has regular three monthly follow-up in breast clinic and is thus far disease free.

## 3 Discussion

The emergence of MPCs in individuals with a history of cancer has garnered increasing attention in medical research, the prevalence of which is between 0.734 and 11.7 %. [4] Studies have indicated that individuals with one primary cancer are at a heightened risk of developing a second primary cancer when compared to the general population. [7] The top- most frequent MPCs were the following: breast; liver; head and neck; colorectal; male genital cancer–prostate; skin; female genital cancer–uterine; thyroid; lung; and female genital cancer–non-uterine [10]. The incidence of MPCs, in general, has exhibited an upward trend in recent years. This upward trend in MPCs may be attributed, at least in part, to advancements such as enhanced imaging capabilities which have led to earlier cancer detection, and innovative treatments which have resulted in improved survival rates. While these advancements are promising, they also raise questions about the relationship between cancer treatments and the development of multiple primary cancers. One prominent risk factor often associated with the development of MPCs is smoking. Notably, her family history is positive for cancer, with her mother having had lung cancer. This familial predisposition raises the possibility of a genetic component contributing to MPCs in this patient. The criteria established by Warren and Gate [1] for identifying MPCs, including the dissimilarity of the neoplasms in pathological analysis and the exclusion of metastases, are fully satisfied in this case. Previous studies have reported a prevalence rate of approximately 13.1 % for synchronous primary breast neoplasia with RCC, primarily involving receptor-positive IDCs [11,12]. Numerous potential etiological factors are being evaluated to understand the pathogenesis of MPCs including, environmental exposures, genetic predisposition, chemotherapy, radiation exposure, hormonal deregulations, and gender-specific elements. MPCs can occur in any age group, but the majority of diagnosed patients are typically older than those with solitary primary neoplasms, with over 75 % being over 50 years of age. Furthermore, a review of the literature revealed that males are more predominantly affected than females with regards to synchronous and metachronous neoplastic occurrences [11]. From a therapeutic perspective, the management of MPCs is complex and often requires coordination among various medical specialties. Our patient was also thoroughly discussed in multidisciplinary team meetings involving, a breast surgeon, urologist, radiologist, oncologists, physiotherapists, and nurses and underwent regional excisions for both IDC and RCC. As we continue to unravel the intricacies of MPCs, documentation of such instances plays a pivotal role in enabling the surgical and epidemiological sectors to institute preoperative screening protocols, advance research endeavors, delineate the frequency of occurrences, and enhance intraoperative surgical methodologies [11]. Breast cancer is the most common tumor to be associated with other primaries especially colorectal cancer, endometrial and ovarian cancer, yet the occurrence of invasive ductal carcinoma with clear cell renal cancer is uncommon. [3] On the other hand, as many as 16–27 % of patients with RCC have other synchronous malignancies [10]. We have yet to identify the exact genetic markers or mutations responsible for these synchronous malignancies. This gap in our knowledge highlights the need for further genetic exploration.

The implications of synchronous IDC and RCC extend far beyond their individual clinical profiles. This unique combination of malignancies carries a potentially fatal prognosis, necessitating a comprehensive understanding of its pathogenesis, diagnosis, and management. By doing so, we can take significant strides towards reducing the associated morbidity and mortality, thus enhancing the quality of care and life for affected individuals.

## 4 Conclusion

This exceptional case emphasizes several critical lessons. Patients with breast carcinoma have a risk of other synchronous primary malignancy. [8,9] Clinicians should maintain a high index of suspicion for synchronous primary cancers, particularly in patients with a familial cancer history. [12]. Documentation is essential to raise awareness which will mitigate the potential morbidity and mortality associated with this exceptional concurrence of conditions. [11] This will contribute to the formulation of comprehensive intra and postoperative patient management guidelines, ensuring patients' recovery and reducing the likelihood of recurrence.

## Consent

Written informed consent was obtained from the patient for publication of the case report and any accompanying images. A copy of written consent is available for review by the Editor-in-Chief of this journal.

## Ethical approval

The nature of this study does not require an ethical approval in our institution.

## Funding

This research did not receive any specific grant from funding agencies in the public, commercial, or not-for-profit sectors.

## Author contribution

Dr. Tarbia Hamid.

Concept, analysis, interpretation of data and, drafting and reviewing.

Final approval of the version to be published.

Agreement to be accountable for all aspects of the work in ensuring that questions related to the accuracy or integrity of any part of the work are appropriately investigated and resolved.

Dr. Fatima Nawaz.

Concept, analysis, interpretation of data and, drafting and reviewing.

Final approval of the version to be published.

Agreement to be accountable for all aspects of the work in ensuring that questions related to the accuracy or integrity of any part of the work are appropriately investigated and resolved.

Dr. Nuzhat Khattak.

Concept, analysis, interpretation of data and, drafting and reviewing

Final approval of the version to be published.

Agreement to be accountable for all aspects of the work in ensuring that questions related to the accuracy or integrity of any part of the work are appropriately investigated and resolved.

Dr. Mian Naushad Ali.

Concept, final approval of the version to be published.

Agreement to be accountable for all aspects of the work in ensuring that questions related to the accuracy or integrity of any part of the work are appropriately investigated and resolved.

Dr. Ramsha Hamid.

Concept, final approval of the version to be published.

Agreement to be accountable for all aspects of the work in ensuring that questions related to the accuracy or integrity of any part of the work are appropriately investigated and resolved.

## Guarantor

The guarantor of this study is Dr. Tarbia Hamid and accepts all responsibility for the publishing of this data.

## Declaration of competing interest

There is no conflict of interest that could be perceived as prejudicing the impartiality of the research reported.
